# Persistent auxiliary microbiome of early novel colonizers in the developing rumen with lasting functional significance

**DOI:** 10.1093/ismejo/wraf252

**Published:** 2025-12-01

**Authors:** Ori Furman, Gil Sorek, Sarah Moraïs, Liron Levin, Omar Eduardo Tovar-Herrera, Sarah Winkler, Itzhak Mizrahi

**Affiliations:** Department of Life Sciences, Ben-Gurion University of the Negev, Beer-Sheva, 84105, Israel; National Institute of Biotechnology in the Negev, Ben-Gurion University of the Negev, Beer-Sheva, 84105, Israel; Department of Life Sciences, Ben-Gurion University of the Negev, Beer-Sheva, 84105, Israel; National Institute of Biotechnology in the Negev, Ben-Gurion University of the Negev, Beer-Sheva, 84105, Israel; Department of Life Sciences, Ben-Gurion University of the Negev, Beer-Sheva, 84105, Israel; National Institute of Biotechnology in the Negev, Ben-Gurion University of the Negev, Beer-Sheva, 84105, Israel; Department of Life Sciences, Ben-Gurion University of the Negev, Beer-Sheva, 84105, Israel; National Institute of Biotechnology in the Negev, Ben-Gurion University of the Negev, Beer-Sheva, 84105, Israel; Department of Life Sciences, Ben-Gurion University of the Negev, Beer-Sheva, 84105, Israel; National Institute of Biotechnology in the Negev, Ben-Gurion University of the Negev, Beer-Sheva, 84105, Israel; Department of Life Sciences, Ben-Gurion University of the Negev, Beer-Sheva, 84105, Israel; National Institute of Biotechnology in the Negev, Ben-Gurion University of the Negev, Beer-Sheva, 84105, Israel; Department of Life Sciences, Ben-Gurion University of the Negev, Beer-Sheva, 84105, Israel; National Institute of Biotechnology in the Negev, Ben-Gurion University of the Negev, Beer-Sheva, 84105, Israel

**Keywords:** early life rumen microbiome, early colonization, rumen microbiome development, microbial succession, temporal dynamics of the microbiome, functional enrichment, microbial adaptation, dietary transitions, metabolic interactions, functional redundancy

## Abstract

The early life assembly of the rumen microbiome is a critical process with lasting implications for host development and function. Using high-resolution longitudinal metagenomics in calves tracked from birth to three years (∼800 days) of age, we reconstructed 2873 high-quality metagenome-assembled genomes, including 517 novel genomes primarily detected in early life. These novel genomes, spanning 274 genera and largely classified as non-core taxa, reveal a diverse and functionally distinct auxiliary microbiome. Unlike in other ecosystems, this early microbial community persists into adulthood, retaining ecological and functional relevance despite a decline in abundance. Temporal clustering revealed strong associations between auxiliary taxa and dietary transitions, with functional enrichments in environmental sensing, nutrient biosynthesis, and volatile fatty acid metabolism. Metabolic network analyses showed that auxiliary genomes complement non-auxiliary community members in key functions, with potential effects on the host. Our findings suggest that early colonizers act as ecosystem engineers, with the potential to shape the developmental trajectory of the rumen microbiome. This study thus positions the early microbiome not as a transient feature of colonization, but as a structured, functionally coherent auxiliary community that interacts with the mature rumen ecosystem.

## Introduction

The question of how complex biological systems develop and function has long fascinated scientists across disciplines. Although we often think of organisms as fully formed entities, the reality is far more difficult. Every living being undergoes a process of development, during which its various systems and organs mature and become functional. This developmental journey is not solely driven by genetics but is profoundly influenced by interactions with the environment, including the gradual colonization by microorganisms [1, 2]. These microbes, although subjected to host filtering and dietary selection, carry functions that are essential for the maturation of host systems and long-term physiological performance [3–6]. The establishment of these microbial communities, particularly in specialized organs, represents a critical yet often overlooked aspect of organismal development. Understanding this process could revolutionize our approach to animal health, productivity, and even human medicine [6–8]. Recent advances have revealed the complexity and importance of host-associated microbiomes, highlighting their role in shaping host physiology, metabolism, and immune function [9].

In ruminants, such as cattle, sheep, and goats, the rumen stands out as a remarkable example of a specialized organ whose function is intimately tied to its microbial inhabitants. This foregut compartment, essential for the digestion of plant material, hosts a diverse and dynamic microbial ecosystem that develops from birth [7, 8, 10, 11]. The rumen microbiome’s assembly is a complex process that begins with early colonizers and progresses through various stages as the animal matures [2, 10, 12, 13, 14, 15]. The result of this assembly process is a stable microbial community characterized by a high degree of functional redundancy and resilience [14, 15, 16, 17]. Despite its crucial role in ruminant nutrition and, by extension, global food production and climate change [8, 18–20], our understanding of the early stages of rumen microbial colonization remains limited, particularly regarding the functional capacities these early colonizers contributed.

The study of these early microbial colonizers is of particular interest due to their potential long-lasting impact on host health and productivity [2]. As in other ecosystems, pioneer microbes in the rumen may play a crucial role in shaping the developing environment and guiding the succession of subsequent microbial communities [8, 18–20]. Colonization history, that is, the timing of microbial species arrival, is known to affect subsequent community assembly [2, 21, 22]. In addition to age, factors such as diet, host genotype, probiotic supplementation, and immune interactions also influence the dynamics and functionality of early colonizers [23–26]. This raises key questions about the functional identity of pioneer microbes, the temporal dynamics of their establishment, and how these early colonization patterns contribute to shaping both the developing and mature rumen environment and its functional capacity. Our study aims to address these knowledge gaps by focusing on the early microbial colonizers in the developing rumen of young calves, tracking their establishment and dynamics from birth through weaning and into adulthood. To investigate their identity, temporal patterns, and functional potential, we leveraged a high-resolution longitudinal metagenomic dataset comprising samples collected over ~800 days, spanning birth to maturity. Using genome-resolved analysis, we reconstructed 2873 high-quality metagenome-assembled genomes (MAGs), including 517 novel genomes that were primarily detected in early life. These previously unreported taxa, spanning over 270 genera and largely absent from existing rumen genome collections, provided a unique opportunity to examine the ecological and functional roles of early colonizers. By analyzing their temporal trajectories and metabolic features, we identified a persistent subset of early auxiliary microbes, distinct from the core microbiome, that exhibited stage-specific functional enrichments, responded dynamically to dietary transitions, and showed genomic potential to complement non-auxiliary genomes in key metabolic pathways.

## Materials and methods

### Animal handling and sample collection

**Figure 1:**
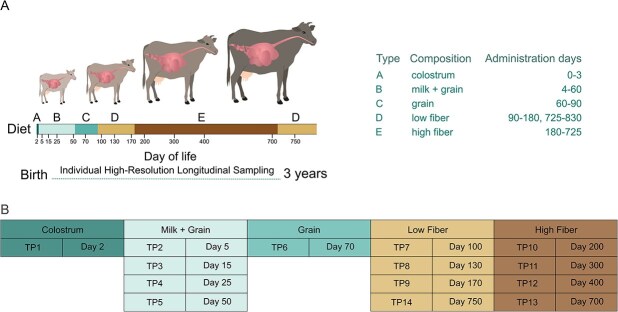
Time-series experimental setup. (A) Dietary regime and sampling points. The experimental setup consisted of 10 cows, sampled in intervals from the second day after birth over a period of 3 years, resulting in high-resolution sampling of 124 metagenomes (bars represent sampling points). The animals were fed with standard dairy-feeding protocols (table on the right), kept under the same conditions, and housed together from the third month of life. (B) Summary of sampling TP within each diet. For convenience, sampling days were categorized into TP (table on the bottom).

The study was conducted at the experimental dairy farm of the Volcani Center, Agricultural Research Organization, Israel, with approval from the local ethics committee (412/12IL, 566/15IL). Animal management, dietary regimes, and rumen sampling procedures were based on Furman *et al*. [2]. Briefly, calves received colostrum for the first 3 days, milk replacer and grain until 60 days, starter feed until 90 days, a low-fiber diet from 90–180 days, and a high-fiber diet until ~725 days, after which cows were returned to a low-fiber ration. Rumen fluid was collected via stomach tube on Days 2, 5, 15, 25, 50, 70, 100, 130, 170, 200, 300, 400, 700, and 750. (Fig. 1A and B).

### Bacterial and DNA extraction

Thawed rumen samples were transferred to centrifuge bottles and kept on ice for no more than 20 min before processing. Rumen samples were processed as described previously [27]. The samples were centrifuged at 10000 × *g* and the pellet was dissolved in extraction buffer (100 mM Tris–HCl, 10 mM ethylenediaminetetraacetic acid, 3% w/v Tween 80, 0.15 M NaCl, pH 8.0); 1 g of pellet was dissolved in 4 ml of buffer and incubated at 4°C for 1 h, as chilling has been shown to maximize the release of particle-associated bacteria from ruminal contents [28]. The suspension was then centrifuged at 500 × *g* for 15 min at 4°C to remove ruptured plant particles while keeping the bacterial cells in suspension. The supernatant was then passed through four layers of cheesecloth, centrifuged (10 000 × *g*, 25 min, 4°C) and the pellets were kept at −20°C until DNA extraction.

### Quality control of metagenomes

Initial quality control of sequencing reads was performed using FastQC (v0.11.8) and MultiQC (v1.0.dev0) [29]. An average of 66.9 M ± 21.4 M paired-reads (Fig. S1A) were generated. Sequences were quality trimmed and filtered using Trim Galore (v0.5; parameters: paired, quality = 25, length = 36) [30] and cutadapt (v1.18) [31]. After trimming, each sample had an average of 66.8 M ± 21.3 M paired-reads with an average sequence length of 147 bp. All samples were then subsampled to 38 M paired-reads per sample using SeqKit, which was the lowest read depth.

### Metagenomic-assembled genomes assembly and binning

Paired-end sequences from each sample were assembled independently into contigs with SPAdes (v3.13; parameters: meta, k-mer = 213 355) [32], and contigs quality was assessed using Quast (v5.0.2), which produced an N50 value of 5.69 ± 4.92 Kbp. The contigs were binned into MAGs using Maxbin2 [33] and Metabat2 [34], followed by bin refinement which consolidates multiple binning predictions into a superior bin set. Binned MAGs were then filtered based on 50% completeness and 10% contamination using CheckM [35], resulting in a total of 7942 MAGs. Among these, a set of 2873 high-quality MAGs was defined, each meeting stringent quality criteria of ≥90% completeness and ≤ 5% contamination (Fig. S2A). Similarity of reconstructed MAGs to rumen-specific genome collections.

**Figure 2:**
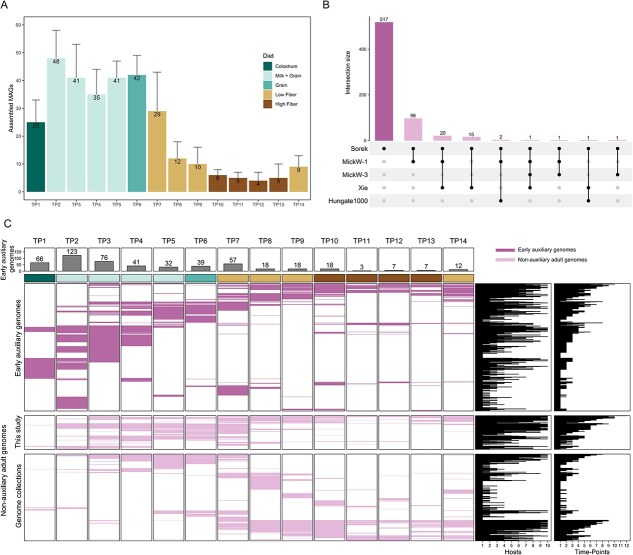
The early auxiliary microbiome. (A) Number of assembled MAGs per host across time. The number of assembled MAGs per host across time (average ± standard deviation, numbers are indicated above each bar), colors representing the diet fed by the host at the time of sampling. Calculations were based on the redundant set (before de-replication) of the 2873 high-quality MAGs (Fig. S2A). (B) Number of species overlapping among rumen-specific genomic collections. The number of species overlapping among rumen-specific genomic collections and our study. Genomes demonstrating ≥95% ANI were considered to be shared between two datasets. (C) Temporal dynamics of early auxiliary genomes and non-auxiliary adult genomes across the sampling period. Genome presence is indicated (true/false) and color -coded for early auxiliary genomes and non auxiliary adult genome, determined by ≥90% coverage of each MAG within a given sample. Bars along the columns represent the number of non-redundant auxiliary genomes assembled at each TP, with numbers indicated above each bar. Bar graphs on the right show, for each genome, the number of unique hosts and the number of TP in which it was detected.

Recent analysis supports a 95% ANI threshold to delineate microbial species [36, 37]. The ANI values of MAGs from our current study (2873 genomes) and genomes from the Hungate1000 project (408 genomes) [38], Stewart *et al*. [39] (2340 genomes), Stewart *et al*. [40] (173 genomes), and Xie *et al*. [41] (1559 genomes) were compared in a pairwise fashion with dRep (v3.4; parameters: P_ani = 0.9, S_ani = 0.95) [42]. All datasets were similarly filtered to include only high-quality MAGs (completeness ≥90% and contamination ≤5%). Genome pairs with ≥95% ANI were denoted as overlapping species between the datasets. We visualized the number of overlapping genomes between each pair of datasets using UpSet from ComplexHeatmap. Our dRep clustering yielded a representative set of 3294 bacterial MAGs and 60 archaeal MAGs, compiled from all rumen-specific genome collections together with those recovered in this study (Tables S1 and S2). Novel genomes were defined as representative genomes of 95% ANI clusters from our study without any overlapping species from other genomic collections/datasets. A total of 517 novel genomes were found according to the above criteria (Fig. 2B).

### MAGs presence inference in metagenomes

Quantification of MAGs within the samples (presence inference) was done by alignment of metagenomic samples using bowtie2 to the MAGs. The read depth was determined using the “pileup” module from BBtools. The abundance values were then normalized to the minimum read depth of the samples, and filtered according to 90% coverage of each MAG within a given sample. Additionally, the percentage of unaligned reads for each was also calculated (Fig. S3).

### Inference of genome trees

Phylogenetic trees were inferred with the representative genomes of all 95% ANI clusters from the dRep analysis (3294 bacterial genomes and 60 archaeal genomes), using the GTDB-Tk [43] (default parameters for the identify, align and infer commands). iTOL [44] was used to visualize the resulting Newick trees and associated metadata.

### Taxonomic and functional annotation of MAGs

Taxonomy was assigned to MAGs using the classify workflow (classify_wf) of the Genome Taxonomy Database Toolkit (GTDB-Tk v2.2.6; parameters: skip_ani_screen) with the associated Genome Taxonomy Database (release 207 v2). In short, the GTDB-Tk classifies each genome based on ANI to a curated collection of reference genomes, placement in the bacterial or archaeal reference genome tree, and relative evolutionary distance. For consistency, genomes from the Hungate1000 project, Stewart *et al*. [39], Stewart *et al*. [40], and Xie *et al*. [41] studies were also assigned taxonomy with the GTDB-Tk [38]. MAGs were functionally annotated with anvi’o (v7.1) [45] according to their metabolic reconstruction vignette. Open-reading frames were annotated into KEGG Orthologs (KOs) [46] using the KEGG database. Pathways completeness was calculated using “anvi-estimate-metabolism”.

### Defining the core microbiome

Core microbes were defined based on the core microbiome framework established by Wallace *et al*. [47]. For this study, core microbes were identified through a combined approach: we first included taxa classified as core in Jami and Mizrahi [48] and Henderson *et al*. [49]. We then incorporated data from the study of Wallace *et al*. [47], defining core taxa as those present in at least 80% of the sampled animals within the above mentioned dataset. The final core list consisted of genera that appeared in at least two out of these three datasets. To assign core attributions to the representative genomes of all 95% ANI clusters from the dRep analysis (3294 bacterial genomes and 60 archaeal genomes), we used the set of predefined core ESVs. MAGs were defined as core using BLAST-based sequence similarity (BLASTn v2.10.1+) between core ESVs (16S rRNA genes) and the MAG sequences. We retained BLAST hits longer than 250 bp (Alignment_Length ≥ 250) with percent identity of at least 90% (Perc_Ident ≥90). MAGs with BLAST hits passing these criteria were defined as core.

### Enrichment analysis

Enrichment analyses were performed by comparing the representation of novel genomes within families, considering the total number of genomes, the number of genomes in that taxon, and the total number of novel genomes to the overall distribution of families across all genera [hypergeometric test, false discovery rate (FDR) corrected *P* < .05].

### Clustering of early auxiliary genomes

To identify groups of genomes with similar temporal dynamics, we constructed a correlation-based co-abundance network using genome-level relative abundance profiles across time. First, we calculated pairwise Spearman correlations between genomes. Correlation values below 0.75 were set to zero to retain only strong positive associations, and any missing values (due to invariant profiles) were also set to zero. The resulting filtered correlation matrix was used to construct an undirected, weighted network, where nodes represent genomes and edges represent strong co-abundance relationships. We applied the Louvain community detection algorithm to identify genome clusters within this network. This clustering information was used in subsequent analyses to track community-level patterns over time.

### Microbial community structure analysis

To evaluate whether microbial community structure was primarily shaped by sampling time point (TP) or by individual animal identity, we calculated Bray–Curtis dissimilarities from the 3354 representative MAGs relative abundance data using the phyloseq package in R (v4.3.0). Ordinations were visualized by Principal Coordinates Analysis with samples colored by TP and distinguished by animal ID using different shapes (Fig. S4). To formally assess the relative contributions of time and host identity, we applied permutational multivariate analysis of variance (PERMANOVA) with 999 permutations using the adonis function from the vegan package (v2.6-4). TP explained nearly half of the variance in community structure (*R*^2^ = 0.47, *P* = .001), whereas animal identity accounted for only a small fraction (*R*^2^ = 0.05, *P =* .045), indicating that temporal progression was the dominant structuring factor.

### Functional pathways completion network analysis

We performed a functional pathway-completion network analysis to evaluate the potential of auxiliary microbiome members to complement and cross-feed the non-auxiliary community. Pathways were reconstructed from the KOs encoded within each genome and mapped against KEGG pathway databases. The functional repertoire of each genome was pairwise compared to all other genomes, and the degree of pathway completion between each two genomes was measured. This analysis performs pairwise comparisons among microbes within a sample to assess whether a pathway identified in one genome can be complemented or completed by another. Input consisted of KO annotation tables generated from genome annotations, and the output included pathway completion scores and pairwise interaction matrices summarizing potential functional complementarity between microbes.

## Results

### Experimental design and data collection

In this study our main goal was to capture the genomic repertoire of early rumen microbial colonizers, which may hold critical functional roles in the rumen but are often missed as they transition into low abundance species. We aimed to characterize the early microbial colonizers of the rumen, exploring their contribution to the establishment and development of the rumen ecosystem. To that end, we utilized samples from our previously published time-series experiment with high temporal resolution (Fig. 1) [2]. From this dataset, we selected 124 metagenomic samples collected from 10 animals that shared uniform dietary regimes, controlled rearing conditions, and highly similar genetic backgrounds (all animals were Holstein–Friesian breed with 75% average genetic similarity). We selected samples spanning the development of their ruminal microbial community from birth over a three-year period, with an average of 12 ± 2 samples per animal (Table S3). Our sampling regime and selected samples involved short-interval sampling during the first months of life, starting from 2-day-old animals and continuing for more than two years (up to 750 days after birth), thereby enhancing the likelihood of capturing the rapidly changing compositions of the early microbiome. This approach also provided high-resolution insights while the ecosystem's complexity remained low. These early life bovine samples, analyzed by shotgun sequencing and MAG assembly, an approach not previously applied at this stage, offer a unique perspective on rumen community assembly, with their low complexity facilitating the recovery of pioneer microbial genomes.

In our experimental design, calves were separated from their mothers immediately after calving and housed in individual kennels at the farm site for the first 90 days. From the third month onward, the animals were co-housed and maintained under similar conditions for the remainder of the study. During each dietary period, the animals were fed with standard dairy feeding protocols according to their age as we previously described (Fig. 1) [2]. To evaluate the robustness of our dataset, we first assessed the effect of time on microbiome structure by examining both presence/absence and abundance patterns of MAGs across the time series. Examining the effect of time on microbiome composition relative to inter-animal variability (Fig. S4) showed that temporal shifts in community structure were highly consistent across animals, reflecting a shared developmental trajectory of the rumen microbiome rather than animal-specific stochasticity. A PERMANOVA model supported this pattern (*R*^2^ = 0.47, *P* = .001), indicating that time explained nearly half of the total variance in community structure, whereas animal identity accounted for only a minor fraction (*R*^2^ = 0.05, *P* = .045) (Fig. S4). For convenience, we refer to the different sampling days as TP. In total, there are 14 TP at which the animals were sequenced, corresponding to five different diets. The average sequencing depth across all metagenomes was 66.9 M ± 21.4 M reads (Fig. S1A). Sequencing depth distribution per-host and per-time-point (Fig. S1B and C, respectively) indicate balanced coverage across hosts and TP.

### Defining the early novel microbiome

To achieve our aim of characterizing the early microbial colonizers in the developing rumen of young calves, we leveraged our high-resolution temporal sampling dataset. Metagenomic assembly and binning of the 124 metagenomes yielded a total of 2873 redundant high-quality MAGs, each meeting stringent quality criteria of ≥90% completeness and ≤ 5% contamination (Fig. S2A). These MAGs exhibited considerable variation in genome size, ranging from 0.88 to 6.39 megabase pairs (Mbp, Fig. S2B), reflecting the diverse array of microorganisms present in the developing rumen ecosystem. The assembly quality was further evidenced by N50 values spanning from 4.9 to 908.9 Kbp, indicating robust contig continuity. On average, each MAG comprised 138 ± 100 contigs (mean ± standard deviation, Fig. S2C, see Table S4). This comprehensive set of high-quality MAGs provides a solid foundation for subsequent analyses of early microbiome members and their potential roles in early rumen development.

When assessing the average number of MAGs per TP for each individual host (Fig. 2A), our results indicate that our effort to capture early microbial colonizers in lower-complexity microbiomes was successful, as most MAGs were assembled from metagenomes originating from earlier, less complex TP. Our findings also clearly show that this phenomenon is uniform across all hosts as suggested by the low standard deviation, further supporting our hypothesis of the genomic potential found in these early TP (Fig. 2A). The lower number of assembled genomes in mature samples is most likely attributable to the greater richness of these communities (as we previously reported for these samples; [2]), as well as the potential presence of larger eukaryotic genomes, most likely protozoa. To characterize the assembled genomes and determine their novelty, we compared the MAGs to other rumen-specific genome collections comprising 16 635 redundant genomes stemming from adult animals, including the Hungate1000 Collection (408 genomes; [38]), Stewart *et al*. [40] (4941 genomes), Stewart *et al*. [39] (913 genomes), and Xie *et al*. [41] (10 373 genomes). Genomes were clustered based on approximate species-level thresholds (≥95% ANI) and the overlap between the MAGs from our study and those in the referenced databases was determined. Approximately one-fifth of the MAGs (517) were found to be novel and not found in any of the genome databases at ≥95% ANI (Fig. 2B). We later assessed the overlap between our dataset and previously published datasets by identifying MAGs that shared ≥95% average nucleotide identity (ANI) with genomes from each study. In total, 96 (3.3%), 15 (0.5%), and 1 (0.03%) of the MAGs in our dataset matched genomes from Stewart *et al*. [40], Xie *et al*. [41], and Stewart *et al*. [39], respectively. We found that the size of the external databases did not correlate with the degree of overlap. For example, Xie *et al*. [41] included over 10 000 MAGs but had only 15 overlapping with our dataset, whereas Stewart *et al*. [40] with less than half that number (4941 MAGs), had 96 overlapping MAGs (Fig. 2B). This relatively low overlap with previously published datasets is most likely driven by biological factors. Our study includes a comparatively large cohort of early life samples, which capture taxa that are either absent or occur at very low abundance in late stages from which most rumen datasets have been generated. This provides a functional perspective on bovine rumen community assembly during early development that, to the best of our knowledge, has not been addressed previously. Moreover, the relatively low complexity of early life samples facilitates improved genome recovery and enables the detection of pioneer microbial colonizers not captured in later-life datasets such as those previously published [41]. In addition, differences in geographical origin between studies may also contribute to the observed variation in community composition. When we quantified the number of genomes detected by read alignment, we found that the number of detectable genomes increased over time, pointing to a rise in richness. This further supports the notion that increased complexity hinders assembly efficiency, highlighting the advantage of lower-complexity samples (Fig. 2A and C).

As aforementioned, our goal was to assess the temporal dynamics of both early novel and adult rumen genomes assembled in our study across the sampling period. Our analysis revealed 517 novel genomes (MAGs), existing only in our dataset, with over 83% of the 517 novel genomes originating from the early stages (TP1–TP7). This analysis revealed a clear pattern, where early novel genomes exhibiting markedly higher abundance at early TP in both the early life and adult microbiomes assembled in this study (Fig. 2C, top and middle heatmaps). By distinguishing between early and adult-assembled MAGs, our approach demonstrated that a substantial portion of the rumen microbiome’s functional potential is established during early life and may persist or become active at later stages. Given their temporal specificity and novelty, we refer to this group of genomes hereafter as the “early auxiliary genomes” whereas the non-auxiliary genomes species will be referred to as non-auxiliary adult genomes, as they are more associated with later stages of life. The progressive decline in their detection over time likely reflects a transition toward a more stable, adult-like microbial community as the rumen microbiome matures.

To investigate this notion of the contribution of the early auxiliary genomes to the overall phylogenetic and functional landscape of the rumen, we performed a comprehensive phylogenetic and functional analysis. This analysis included representative genomes from all rumen-specific genome collections, alongside those recovered in the current study. We performed de-replication using dREP [42] on the entire MAG cohort, comprising MAGs from all the aforementioned datasets as well as our own. This process yielded 3294 bacterial MAGs and 60 archaeal MAGs (Tables S1 and S2).

**Figure 3:**
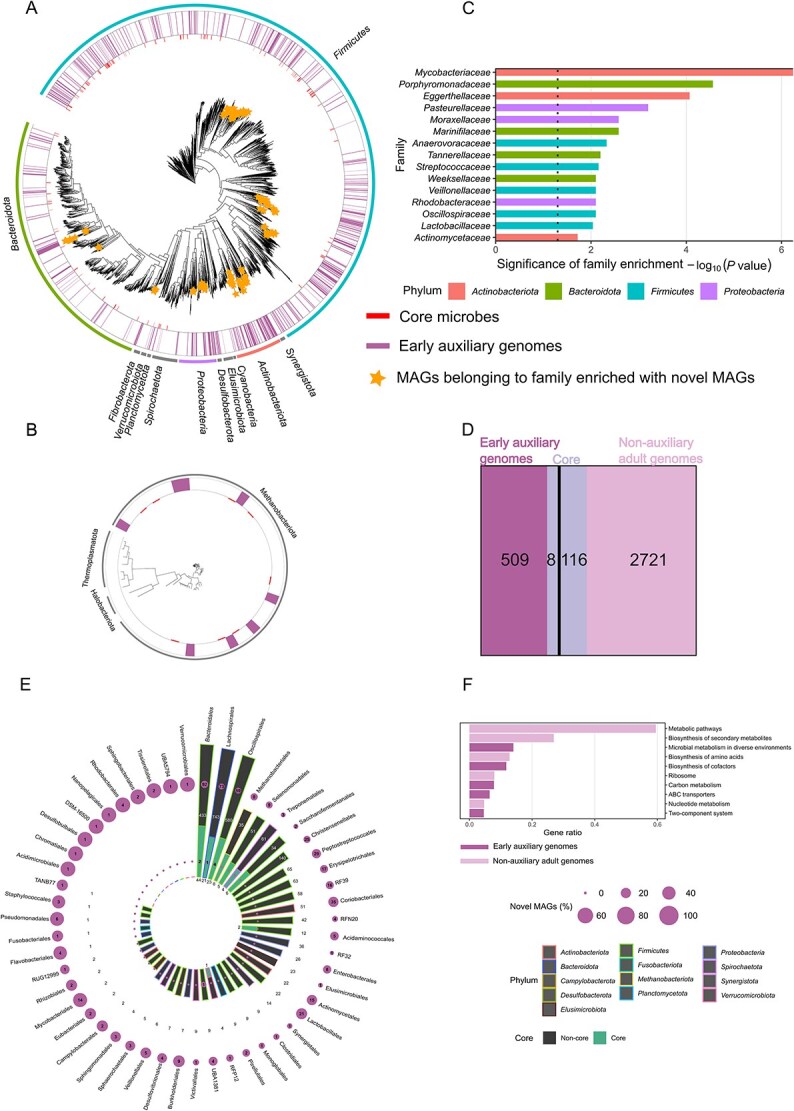
Early auxiliary genomes expand known rumen microbial diversity. (A) Phylogenetic tree of 3294 bacterial MAGs. A maximum likelihood tree representing phylogenetic analysis of 3294 high-quality (≥90% complete, ≤5% contamination) bacterial MAGs was constructed using fastree [88]. These MAGs are the representative MAGs of all 16635 genomes across all datasets, selected using the dREP tool [40]. Stars denote MAGs belonging to family-level taxonomies of early auxiliary genomes. Inner lines indicate core microbes (see Methods: Defining the core microbiome), while mid lines indicate early auxiliary genomes. Outer strips represent the different phyla comprising the tree. (B) Phylogenetic tree of 60 archaeal MAGs. 60 high quality archaeal MAGs inferred from the concatenation of phylogenetically informative proteins. Inner lines surrounding the tree denote core microbes and mid lines denote early auxiliary genomes. Outer strips represent the different phyla comprising the tree. (C) Families enriched with early auxiliary genomes. Families that are enriched with early auxiliary genomes, as determined by comparing their frequencies to the null model (hypergeometric test, FDR corrected *P* < .05). Colors represent the phyla taxonomy level of the enriched family. (D) Auxiliary–non-auxiliary and core relationships. Venn diagram of 3354 high-quality bacterial and archaeal MAGs from rumen-specific genomic collections and the current study. The analysis reveals minimal overlap between auxiliary (Left rectangle) and core (mid rectangle) MAGs, with core genomes being more prevalent within the non-auxiliary rumen microbiome (right rectangle). Core assignments were based on 16S rRNA gene BLAST searches against genome references. (E) Auxiliary-to-core taxonomic relationships. Comprehensive auxiliary-to-core taxonomic relationships of auxiliary and shared adult taxa from published genomic collections and those identified in this study at the order level. Bars represent genomes from published genomic collections, (absolute numbers within the inner and outer circle; bar heights shown on a log scale), divided into core and non-core categories. Circles represent early auxiliary genomes from the current study, categorized as core (positioned in the bottom portion of the bar) or non-core (positioned in the top portion). Circle size corresponds to the percentage of early auxiliary genomes expanding existing genomes at the order level, with absolute numbers indicated within each circle. Colors surrounding each bar denote phylum-level taxonomy. (F) Enriched functions on early auxiliary genomes and non-auxiliary adult genomes. Functions enrichment test was performed using a hypergeometric test, with a threshold of P < .05 (FDR corrected), top 10 enriched functions were chosen in this analysis gene ratios represent the prevalence of the pathways within each group.

Phylogenetic trees of representative bacteria and archaea (Fig. 3A and B, respectively) reveal that the early auxiliary genomes are distributed across 274 out of 813 genera. The appearance of a third of all rumen genera early on in life may suggest the onset of a positive selection pressure, this is further corroborated by their ongoing persistence within the rumen (Fig. 2C), indicating a deterministic event rather than a casual inoculation. Microbial families enriched with early auxiliary genomes (Fig. 3C) further elucidates this trend of highly adapted microbial genomes, primarily within the *Actinobacteriota*, *Bacteroidota*, *Firmicutes*, and *Proteobacteria* phyla. Enrichment of novel *Proteobacteria* genomes was observed during the earliest stages of rumen development, prior to the transition to anaerobic conditions [10, 50]. Hence, the high taxonomic diversity of our early auxiliary genomes across multiple genera argues against a scenario of opportunistic colonization. Instead, it highlights the early establishment of a complex microbial system and underscores the interdependent relationship between host and microbiome. We propose that early life positive selection and habitat sorting drives this early life composition.

### Early auxiliary genomes expand the non-core rumen auxiliary microbiome and contribute distinct functional traits

We next set to understand where the early auxiliary genomes are situated within the rumen microbiome hierarchy with regard to prevalence, taking into account the core microbiome concept which essentially defines microbes according to their degree of prevalence across individual hosts, where core microbes are highly prevalent across hosts and non-core are less [16]. To evaluate how the early auxiliary genomes align with the established core–non-core framework, we examined its distribution across previously defined core genera, taking into account the dataset of Wallace *et al*. [47] as well as other studies describing core rumen microbes [49, 51] (see Methods section: Defining the core microbiome). Using these datasets, we identified core genera based on annotations and ESV alignment to the MAGs (see Methods section: Defining the core microbiome). Out of the 517 early auxiliary genomes identified, only 8 were found to be within core genera, whereas the remaining 509 were classified as non-core (Fig. 3D). Our analysis revealed that early microbiome genomes predominantly expand the *non-core* fraction of the rumen microbiome. Rather than reinforcing the established core community, they introduce novel genomes from orders enriched specifically during early life, many of which likely harbor previously uncharacterized functions. To gain deeper phylogenetic insight into the extent to which these early auxiliary genomes expand our understanding of the gene content and taxonomic landscape of the rumen microbiome, we asked whether they belong to entirely distinct phylogenetic orders, or whether they represent subsets of known core-associated lineages at the genus level that simply emerge earlier in life, and if so, to what extent. To address this, we examined taxonomic affiliations up to the order level. For each order represented by core or non-core genomes in our dataset and previously published collections, we quantified the number of early auxiliary genomes classified as core and non-core members. This analysis identified ten taxonomic orders absent from all previously reported rumen-specific genome collections, to which our study contributed fifteen early auxiliary genomes (Fig. 3E). To further investigate the implications of the early auxiliary genomes, we examined their functional repertoire in comparison to shared adult taxa by identifying functions significantly enriched in each group (Fig. 3F; see Methods). Among the ten most enriched functions, five were associated with early auxiliary genomes and five with the non-auxiliary adult genomes, indicating a clear functional differentiation between these groups. This suggests that early auxiliary genomes not only broadens our understanding of rumen microbial diversity but also contributes distinct functional capabilities to the ecosystem. Functions such as *biosynthesis of cofactors*, involved in the production of essential vitamins and cofactors that support diverse metabolic pathways, were more prevalent in early auxiliary genomes [38, 52]. For example, two vitamins enriched in this group have been shown to influence both rumen microbes and animal productivity. Specifically, cobalamin (vitamin B12) supplementation has been reported to increase propionate production by the rumen bacterium *Prevotella ruminicola* [53, 54]. In addition, supplementation of folic acid to lactating cows showed increased performance in milk yield [55]. This notion is further supported by studies showing that diets with higher grain content are associated with enhanced vitamin biosynthesis [56], consistent with the dietary regime provided to our animals from TP 7 onward. Additionally, ABC transporters (Fig. S4) and two-component system, both enriched in early auxiliary genomes, have been previously linked to dietary adaptation and microbial environmental sensing in ruminants [57–60]. ABC transporters, which are known to mediate the uptake of plant-derived sugars and oligosaccharides via specific membrane-bound proteins [61], have been linked to different diets and dietary components. For example, early life feeding of goat kids with maternal milk led to an upregulation of this function over ones fed with milk replacer [62]. Moreover, up-regulation of ABC transporter system proteins was documented in the rumen bacterium *Butyrivibrio proteoclasticus* when grown in vitro on xylan [61]. Indeed, two of the novel genomes identified in our study belong to the *Butyrivibrio* genus, making it plausible that their regulation reflects a direct response to the dietary changes occurring during development. Most of the early auxiliary genomes members were detected during the pre-weaning period, a developmental window characterized by constant dietary transitions as calves gradually increase their solid feed intake in parallel with rumen maturation. It is therefore reasonable to assume that the functional enrichments observed in these early colonizers confer adaptive advantages that facilitate their establishment and persistence during this dynamic phase of community assembly and host development. Together, these findings suggest that early auxiliary genomes harbor functional traits that may play key roles in the initial establishment and adaptation of the rumen ecosystem, potentially influencing host development and microbial succession.

### Early auxiliary genomes are functionally significant throughout life

Having established the contribution of early auxiliary genomes to the functional diversity of the early rumen microbiome, we next sought to investigate their ecological significance and temporal dynamics across host development. Specifically, we aimed to determine if the prevalence and functional importance that the early microbiome shows over time, potentially correlates with key developmental stages or environmental changes. Embedded in this approach was the question of whether the early novel microbiome is limited to early rumen development or continues to play a significant role throughout the host’s life.

**Figure 4:**
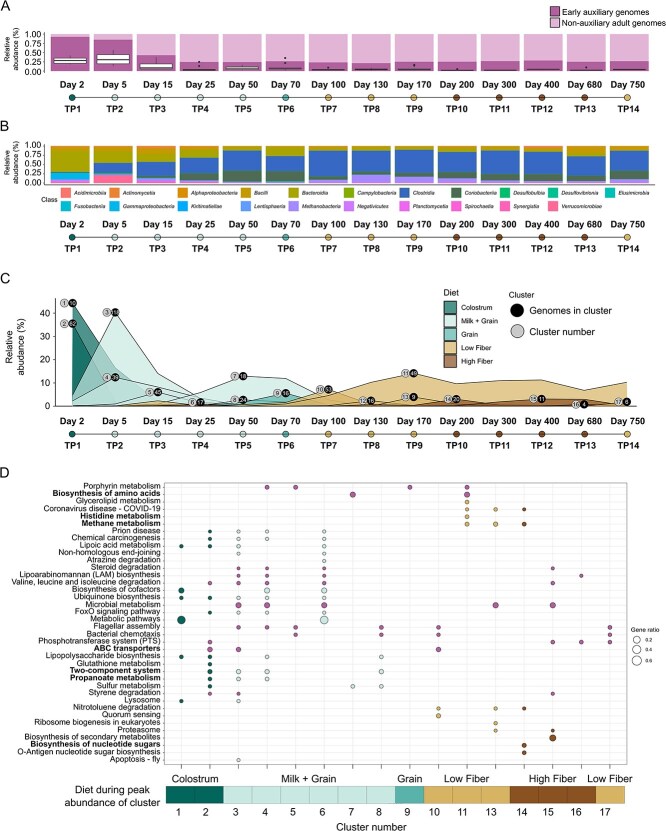
Diet- and age-dependent clustering shows persistent functional roles of early auxiliary genomes. (A) Total relative abundance occupied by early auxiliary genomes is higher at early time points (TP) but remains constant throughout life. Summary of total relative abundance occupied by the early auxiliary genomes and non-auxiliary adult taxa across time (*X*-axis) and all animals. Total microbial abundance refers to the sum of coverage of the entire MAG collection at a given TP across animals, which is then normalized to 100%. Boxplots show the percent coverage of the most abundant microbe within the early auxiliary genomes across all samples within each time-point. (B) Relative abundance (class level) distributions across time of only the early auxiliary genomes. Relative abundance distributions across time (*X*-axis) of only the early microbiome at the class level within each TP. (C) Early auxiliary genomes are clustered according to age and diet. Clustering analysis of the early microbiome based on abundance profiles over time. Shown are 17 distinct clusters, with the right circles highlighting the peak of total relative abundance (Y values) occupied by the genomes in the cluster, together with the number of genomes in the cluster (numbers inside the circles). Left circles denote the cluster number. The colors of the area under the curve denote the corresponding diet fed with the time-point at which the cluster was at its peak abundance. (D) Enriched functions in each microbial cluster of early auxiliary genomes. Functional enrichment of clusters is age- and diet-dependent. An enrichment analysis was performed on the functional profiles of all clusters (hypergeometric test, FDR corrected *P* < .05), finding enriched functions in 16 out of the 17 identified clusters (also see Methods). Each dot represents a function enriched within a given cluster: Dot size reflects the gene ratio (number of genes associated with the function relative to the total). Functions are shown on the *Y*-axis and cluster numbers on the *X*-axis. The colored bar along the bottom of the plot indicates the predominant diet during the peak abundance of each cluster as depicted in section C. Functions enriched exclusively in milk-supplemented diets (colostrum, milk + grain, grain) or in fiber-based diets (low Fiber, high Fiber) are colored according to the corresponding diet.

To address these questions, we analyzed the relative abundance of the early auxiliary genomes across all samples and TP by aligning reads to each MAG and calculating the fraction of metagenomic reads mapping to that MAG relative to the total reads in the entire metagenome within each TP (Fig. 4A). The results revealed a temporal pattern in the early microbiome’s abundance. Initially, the early auxiliary genomes dominated the rumen ecosystem, accounting for approximately 95% of the total microbial abundance, where total microbial abundance refers to the sum of coverage of the entire MAG collection at a given TP, which is normalized to 100%. This high initial prevalence suggests a critical role for early auxiliary genomes in the early establishment of the rumen ecosystem. However, by Day 25 (TP4), the early microbiome's total relative abundance decreased to 25%–30%, where it remained relatively constant throughout the rest of the host's life. This pattern indicates a shift from an early dominated to a more diverse microbial community as the rumen matures. During this period, the diet remains relatively stable (Fig. 1A and B), making shifts in the microbiome more likely to be driven by host developmental changes, such as the onset of rumination and the gradual increase in dry feed intake alongside milk consumption during the first month of life. In addition, the introduction of new species from the herd’s gamma-diversity pool could create competition and reduce the abundance of resident species. Hence, although host-driven changes are likely the dominant factor in species filtering, contributions from competitive dynamics introduced by new colonizers, facilitated by changes in habitat, are also likely to play a role. Box plots for each TP illustrate the relative abundance of the most prevalent early auxiliary genomes across all samples within a specific TP (Fig. 4A). These observations suggest that individual early auxiliary genomes maintain relatively high abundances at earlier TP, indicating that our ability to identify these microbes in later stages, when their total relative abundance is high is not merely due to a larger number of low-abundance organisms, but rather because each microbe individually occupies a significant niche in the ecosystem. While the overall abundance of the early microbiome stabilized after TP4, a closer examination at the class level revealed ongoing compositional changes (Fig. 4B). These findings suggest that, although members of the early microbiome do not necessarily persist over time, they may still retain functional relevance in the rumen by adapting to shifting environmental conditions or host developmental stages. To further investigate the drivers of these compositional shifts, we clustered the early auxiliary genomes based on their abundance profiles over time, resulting in 17 distinct clusters, with approximately 40% of the bacteria and archaea in these clusters being classified at the species level (Tables S1 and S2). Among these 40%, many genera and species carried barcoded names (for example–RGIG4310, GCA-900199385), highlighting how poorly characterized this microbial community remains. Visualization of these clusters' relative abundances revealed that their dynamics closely corresponded to dietary changes throughout the host's life (Fig. 4C). We next sought to identify the functional potential associated with each cluster and relate it to the temporal dynamics characterizing their trajectories. To this end, we performed an enrichment analysis to determine which functions were significantly overrepresented in each cluster (see Methods), identifying a total of 38 enriched functions (Fig. 4D). Functional enrichment patterns of the clusters exhibited temporal dynamics that, while not highly specific, were nonetheless closely aligned with dietary shifts, despite distinct taxonomic compositions between clusters (Fig. 4C). For example, functions such as biosynthesis of nucleotide sugars, histidine metabolism, and methane metabolism were enriched at TP corresponding to fiber-based diets (TP7–14), whereas functions including propionate metabolism and two-component systems were enriched during milk-related dietary stages (TP1–5) (Fig. 4D). We observed that clusters 1 to 6, although taxonomically distinct, each peaked in relative abundance during the first month of life and shared a common set of 25 enriched functions. Consistent with the independent enrichment analysis (Fig. 3F), vitamin biosynthesis pathways identified as enriched in early auxiliary genomes were also found here to be enriched at TP 1, 4, and 6. The biosynthesis of vitamins in grain and fiber-fed newborn calves has been documented as early as 1954 [63], and increased expression of vitamin and cofactor biosynthesis has more recently been reported in response to grain-containing diets [56]. These observations are consistent with the examined stages, where animals were already receiving solid feed containing both grain and fiber (Fig. 1). Functional enrichment dynamics of ABC transporters, which have been associated with multiple dietary regimes, were observed in both early and adult diets [61, 62]. Notably, when we calculated the average copy number normalized by genome abundance (Fig. S5), we found that ABC transporters clearly differentiate the early auxiliary microbiome from the non-auxiliary adult microbiome (Fig. S5). This finding underscores their importance during early life and highlights their sustained functional contribution across all stages of rumen development.

The variability in taxonomic composition alongside functional convergence highlights the functional coherence of the clusters during early rumen development and suggests their adaptability and compatibility with the emerging ecosystem structure and function (Fig. 4D). Moreover, our findings align with the concept of rumen functional redundancy in which different taxa can fulfill similar ecological roles, allowing for community stability despite environmental fluctuations [15]. In terms of taxonomic composition, clusters exhibited distinct profiles, with most genera appearing exclusively in a single cluster (Table S1). These clusters harbored genera like the facultative anaerobe *Streptococcus* known as early colonizers of rumen, which convert rumen to a fully anaerobic environment to promote the rapid establishment of strictly anaerobic bacteria [10, 64]. We also detected high prevalence of *Prevotella* and *Alloprevotella* which are also known as early colonizers [10, 59, 65–67]. The presence of this genus highlights the importance of these early auxiliary genomes. *Prevotella* is a versatile microbe that degrades peptides and polysaccharides, producing propionate, a hydrogen sink that may influence rumen methane production [68]. A functional analysis of the *Prevotella* genus (Table S5) revealed that it is highly engaged in metabolic activity. We have observed that *Prevotella* was the only genus prevalent in eleven distinct clusters (Table S1), highlighting both its functional versatility across species and the metabolic significance of these largely uncharacterized *Prevotella* members within the rumen environment [68]. Another member of these clusters is *Akkermansia muciniphila*, whose growth has been reported to be influenced by the presence of bovine colostrum, which was available during the early stages [69]. The onset of such “adult-like” taxa at very early TP, including *Prevotella* and *A. muciniphila*, may be linked to the early introduction of dietary fibers, offered to calves as soon as two days after birth, together with exposure to maternal fecal matter. These factors could facilitate the establishment of these species, consistent with previous findings by Jami and colleagues [10].

The early life clusters were functionally dominant, collectively accounting for 65% of all enriched functions identified. In contrast, clusters 7 to 9, although also apexing within the same dietary period, exhibited substantially fewer enriched functions (only seven). This pattern aligns with previous findings in these same animals, which demonstrated a significant, age-dependent restructuring of the microbial community within this dietary phase [2]. In cluster 11, a prolonged peak in relative abundance was observed, extending across the fibrous dietary stages (Fig. 1 and Fig. 4D). This cluster was composed of genera like *Saccharofermentans*, which has been reported previously in adult cows and is known to convert glucose mainly into acetate, lactate, and fumarate [70]. In addition, the genus *Porcincola*, more commonly found in pigs, was detected among the analyzed samples. It has been characterized as being able to degrade starch and cellulose, thus possibly explaining its presence in this cluster [71]. This genus appears in overall four clusters and it harbores the Wood–Ljungdahl pathway, a known H sink [72]. The presence of the *Bulleidia* genus was also detected, this genus was positively correlated to volatile fatty acid (VFA) production and milk yield in lambs and lactating cows [73, 74], indicating the metabolic contribution of early auxiliary genomes. Of its six enriched functions, three were directly associated with VFA production: biosynthesis of amino acids, histidine metabolism, and methane metabolism. These functions are tightly linked to VFA generation and represent key metabolic processes that support the dietary and energetic requirements of adult ruminants during fiber-rich feeding [75, 76]. We noticed that the transition to a high-fiber diet, as represented by cluster 14, was marked by the enrichment of biosynthesis of nucleic sugars. A comparable response was observed in animals fed hay, which is a highly complex carbon source instead of silage which is considered as more digestible. Suggesting a microbial response to this major change in carbon source [77]. This cluster showed the presence of the butyrate-producing genera *Copromorpha* and *Butyrivibrio* [78, 79]. As this cluster peaks during the transition to a very fibrous diet, it is possible that the presence of the two *Butyrivibrio* species could be linked to this genera’s ability to degrade complex carbon sources [80]. Cluster 12 was detected exclusively at TP 8, which coincided with a relatively low-fiber, high-concentrate dietary phase (Fig. 4C). Our analysis showed that this cluster exhibited no significantly enriched functions. This absence may reflect the reduced dietary complexity during this period, potentially limiting the need for specialized microbial capabilities. The genus *Limimorpha* was also identified in this cluster; this genus was recently reported to possess numerous antiviral defense mechanisms [81]*.* In conclusion, functional enrichment profiles revealed clear temporal and dietary signatures across clusters, reflecting a dynamic interplay between microbial succession and functionality and host nutritional shifts throughout early development. These findings support the hypothesis that the early microbiome is not only important during early rumen development but also continues to contribute significantly to rumen function throughout the host's life. The initial high abundance of early auxiliary genomes may reflect their importance in establishing the rumen ecosystem and preparing it for more complex functions [82]. The subsequent stabilization in overall abundance, coupled with ongoing compositional changes, suggests that the early microbiome plays a role in maintaining rumen plasticity and adaptability to changing dietary conditions [3]. This dynamic behavior was previously observed in ruminants where different microbial communities retained host performance [83].

### Early auxiliary genomes expand rumen metabolic capacity with potential effects on the host

**Figure 5:**
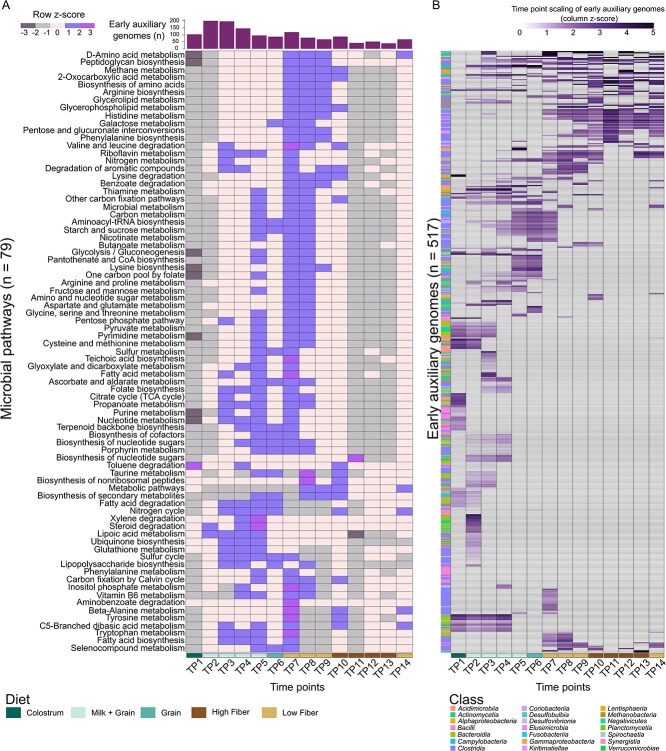
Early auxiliary genomes contribute to the functional completeness of non-auxiliary adult genomes across developmental stages. Functional pathway completion network analysis was performed to assess how early auxiliary genomes complement the metabolic potential of non-auxiliary adult genomes. Genomes were converted into metabolic networks, and interactions were quantified by measuring the extent to which metabolites encoded by early auxiliary genomes completed or expanded pathways in non-auxiliary genomes, calculated as the number of new metabolites produced by the non-auxiliary adult genomes through these interactions (see Methods). (A) Early auxiliary genomes complete central metabolic pathways across all TP. Heatmap showing contributions of early auxiliary genomes to the completeness of non-auxiliary adult genomes across microbial metabolic pathways. Each row represents a distinct pathway, and each column a TP. Completeness values were averaged by dividing by the number of contributing early auxiliary genomes at each TP, and rows were z-score scaled. The bar plot above the heatmap shows the number of contributing early auxiliary genomes per TP. Diet regimes are indicated below the heatmap (colostrum, milk + grain, grain, low fiber, high fiber). (B) Early auxiliary genomes persist into late stages with variable functional contributions. Heatmap showing the degree of contribution of individual early auxiliary genomes to the completeness of non-auxiliary genomes across TP. Each row represents an early auxiliary genome, and each column a TP. Color shading indicates relative contribution levels, normalized within each TP (z-score scaled) to highlight the hierarchy of genome contributions. Rows are clustered by similarity in contribution profiles. Taxonomic classification at the class level is depicted by different colors (right panel). Diet regimes are indicated below the heatmap.

To explore the potential functional contributions of early auxiliary genomes to the rumen ecosystem, and potentially to the host, we performed an analysis quantifying the extent of cross feeding that early genomes could provide to the rest of the community across developmental TP. Our results show that early auxiliary genomes hold substantial potential to support and expand the metabolic capacity of the rumen ecosystem well into adulthood, with possible effects on the host. Specifically, we assessed the presence and absence of both early auxiliary and non-auxiliary adult genomes across time (by read alignment; see Methods and Fig. 2), converted genomes into metabolic networks, and asked to what extent early auxiliary genomes expand the metabolic repertoire of non-auxiliary adult genomes by identifying pathways complemented by early auxiliary genomes and quantifying the additional metabolites produced by these complementations (Fig. 5A). This analysis revealed that early auxiliary genomes consistently complemented non-novel adult genomes across a wide range of pathways (*n* = 79; Fig. 5A). Many of these pathways were complemented by multiple early auxiliary genomes, indicating a high degree of functional redundancy. Several pathways are central to microbial metabolism and have potential consequences for the host. For example, many of the expanded pathways were related to amino acid and vitamin biosynthesis. Amino acids serve as precursors for short-chain fatty acid production [75, 76], a major energy source for the host, suggesting that early auxiliary genomes may influence both microbial interactions and host energy balance. Another example is the lipoic acid metabolism pathway, which was complemented by early auxiliary genomes (Fig. 5A). Exogenous supplementation of this compound has been shown to enhance milk production in dairy cows [62], highlighting the potential for early novel microbes to affect host productivity. We found that the contributions of early auxiliary genomes were not confined to the earliest developmental stages. A substantial proportion reached their highest levels of contribution after TP 7, coinciding with the introduction of a high-energy diet containing ~30% fiber (Total Mixed Ration diet, commonly used in dairsy cattle). The increase in contributions during this dietary transition suggests that early auxiliary genomes act as active functional partners, rather than residual colonizers, helping to shape the metabolic potential of the maturing community. While their contribution declined during the switch to a high-fiber, low-energy diet, it rose again after calving and at the onset of lactation, when animals were reintroduced to a high-energy diet.

To further dissect the patterns observed in the current analysis, we examined the individual contributions of each of the 517 early auxiliary genomes across the entire time series by ranking their relative contributions within each stage (Fig. 5B). This genome-resolved analysis revealed that some early auxiliary genomes contributed only transiently during early life, while others persisted and continued to complement non-auxiliary adult genomes later in development. Of the 517 genomes, 120 (23%) were detected as contributors after Day 100 of life (TP 7, onset of the adult fibrous diet) in at least two later stages, up to the age of 750 days (TP 14) with many of these also ranked among the top contributors within their respective TP. Clustering highlighted distinct groups of genomes with similar temporal contribution profiles, suggesting functional guilds that co-establish during specific developmental windows and dietary regimes. Taxonomic annotation further revealed that contributors were phylogenetically diverse, spanning multiple phyla (Fig. 5B).

Together, our results suggest that early auxiliary genomes are not simply transient colonizers but integral players that cross-feed, expand, and sustain the metabolic potential of the rumen microbiome. Their contributions are characterized by functional redundancy (Fig. 5A), in agreement with our findings of their redundant functional potential (Fig. 4), and they likely play a key role in the long-term metabolic resilience of the rumen ecosystem and its interface with the host.

## Discussion

The early life stages of ruminants represent a critical window during which the foundations of the rumen microbiome are established. In this study, we leveraged a high-resolution longitudinal sampling design to characterize the genomic and functional landscape of the early rumen microbiome, capturing pioneer microbial colonizers and assessing their long-term ecological and functional significance.

Our experimental design, which leveraged a previously established time-series cohort [2], provided uniquely high temporal resolution of the functional capacity of the bovine rumen microbiome during the earliest months of life, a developmental window characterized by rapid microbial turnover and low community complexity, through to mature stages. This work provides a first glimpse into the functional capacity and ecosystem relevance of both early and late developmental trajectories in cattle, offering a comprehensive view of long-term ecosystem assembly and underscoring the immense potential of studying rumen microbiome development from birth to adulthood. In doing so, our study extends and complements other temporal datasets: for example, those focused on later developmental stages and their predictive value for productivity in cattle [14], or those examining non-bovine ruminants only early life without integration into adult stages [84, 85].

A central outcome of our study was the identification of 517 early auxiliary genomes, nearly 20% of the assembled genomes, which were absent from existing rumen-specific genome collections, likely due to their occurrence in early rumen stages that are poorly represented in current databases. These genomes were predominantly associated with early TP, and phylogenetic analysis revealed their wide distribution across 274 genera, thus expanding the known microbial diversity of the rumen. Furthermore, 10 orders containing 15 MAGs were absent from previous large-scale efforts, such as the Hungate1000 [38] and the two datasets from Stewart *et al*. [39, 40] and Xie *et al*. [41], suggesting that early life sampling could uncover hidden microbial diversity. Some of these genomes may not have been assembled previously, even though they likely exist in mature rumen samples, due to technical factors such as limited sequencing depth, the high complexity of mature rumen metagenomes, and the relatively small number of studies targeting the early life rumen microbiome. Thus, while database and methodological biases may partially explain why these genomes were not recovered before, our results underscore that early life sampling provides unique access to this hidden microbial diversity. With increasing efforts, particularly those focusing on early developmental stages, it is likely that additional such genomes will be discovered. Most of the early auxiliary genomes were classified as non-core taxa, a pattern that may partly reflect the predominant focus of core microbiome studies on adult rumen stages. Moreover, their limited prevalence across hosts may stem from the high degree of functional redundancy and ecological equivalence they encompass. While such redundancy may allow these taxa to alternate across individuals, they nonetheless retain ecological importance by fulfilling unique roles within the community. Functional analysis further demonstrated that the early auxiliary genomes harbored enriched pathways, such as biosynthesis of cofactors, ABC transporters, and two-component systems, which are traits likely advantageous in adapting to rapidly shifting environments and dietary transitions [57, 58]. Beyond their metabolic roles, in Firmicutes, certain ABC transporters and neighboring two-component systems have coevolved into tightly linked detoxification modules that both sense and confer resistance to antimicrobial peptides [86]. It is therefore possible that some of the enriched functions we observed are not solely diet related but may also provide these microbes with a competitive advantage. Furthermore, although ABC transporters have reported to be upregulated during microbial exposure to milk [62, 87], yet, its enrichment in TP 10, over a month after weaning, suggests other possible functions. Our longitudinal analysis revealed a clear temporal dynamic in the relative abundance of the early microbiome. Early auxiliary genomes dominated the microbial community immediately after birth, comprising ~95% of total abundance, before stabilizing at ~25–30% by Day 25. Despite this reduction, these early microbes maintained consistent representation throughout the host’s life, suggesting persistent ecological relevance. This trend was not due to the accumulation of many low-abundance taxa; rather, individual early microbes maintained substantial abundance at early TP, indicating niche dominance.

Clustering of MAGs based on their temporal abundance profiles revealed 17 distinct clusters, many of which corresponded closely with dietary transitions. Early life clusters [1–6], which peaked during the first month, shared 25 enriched functions and accounted for 65% of all functions identified in the enrichment analysis. In contrast, clusters peaking slightly later showed reduced functional enrichment, highlighting a potential division between primary colonizers and transitional taxa. Cluster 11 (see composition in Table S1) exhibited a prolonged peak throughout the fibrous dietary stages and included functions directly tied to VFA production, such as histidine metabolism and methane metabolism. These functions are key to rumen fermentation and energy harvesting in adult animals [75, 76]. Furthermore, cluster 14 showed enrichment for nucleic acid biosynthesis upon transition to a high-fiber diet, a trend also observed under hay-based feeding, likely reflecting microbial adaptation to complex carbon substrates [77]. The early auxiliary genomes identified in this study highlight a largely unexplored component of the rumen microbiome. Only ~40% could be annotated at the species level, and many of these remain poorly described, often represented by barcoded names, underscoring the taxonomic and functional gaps that persist in current reference databases. Despite these taxonomic annotation limitations, there are clear signs of their ecological relevance. For example, The *Prevotella* genus includes more than 50 characterized species that occur in varied hosts. Members of this genus are characterized by their versatile metabolic capabilities and their ability to utilize a broad range of substrates including peptides, proteins, monosaccharides, and plant polysaccharides [57, 68, 88]. In our study, this genus appears in eleven clusters, which was the highest number of clusters any genus appeared in, across multiple clusters spanning various dietary phases and developmental stages. Known for its role in plant biomass degradation and its interactions with other fiber degraders, *Prevotella* may act as a key facilitator in these processes [68, 89]. Our functional analysis confirmed that this genus is indeed potentially involved in various metabolic processes throughout time (Tables S5 and S6). The fact that in such a dynamic environment, during major dietary changes, early auxiliary genomes retained at least 20% of the abundance over time strongly suggests that it holds a competitive edge. The presence of other genera such as *Limimorpha* or *Porcincola*, harboring extensive viral resistance and having the potential of serving as alternative hydrogen sink [71, 81], shows us the great potential residing within these unexplored microbes, which warrant deeper investigation. By analyzing the potential metabolic contributions of the auxiliary early genomes to the rumen ecosystem, our findings suggest that these bacterial and archaeal genomes are not merely transient colonizers but remain active contributors to the functional potential of the community throughout development, with important implications for the host. By complementing non-auxiliary adult genomes in key pathways, including amino acid and vitamin biosynthesis, these genomes may support the establishment of a metabolically resilient microbiome and the production of metabolites essential to the host. Their continued contributions following dietary transitions, such as the shift to a low-fiber, high-energy diet and later at the onset of lactation, further indicate that early colonizers play key functional roles under changing nutritional conditions. The persistence of over 23% of novel early genomes as contributors at later TP highlights their potential long-term ecological significance and suggests the existence of functional guilds that help sustain rumen metabolism across life stages. Altogether, these results emphasize that the early microbiome is not only fundamental for initial rumen development but also exerts long-term functional influence. Its persistence, compositional plasticity, and functional contributions across dietary stages support the view that early colonizers may not be transient but integral components of the mature rumen ecosystem.

## Conclusion

Our findings demonstrate that early life microbial colonizers are pivotal to rumen assembly and diversity. They are not merely participants in ecosystem maturation but constitute an integral part of the mature rumen microbiome. Through high-resolution sampling and genome-resolved metagenomics, we uncovered hundreds of novel, predominantly non-core genomes that persist across developmental stages and potentially play functionally distinct roles in the rumen ecosystem throughout host life. These early life microbes were enriched in functions related to nutrient biosynthesis, environmental sensing, and fermentation pathways, and have the potential to metabolically support and complement the rest of the ecosystem with central metabolites, traits that likely facilitate both early colonization and long-term ecosystem performance. As such, they should be considered critical targets in future microbiome-based interventions aimed at improving ruminant health, productivity, and environmental sustainability. For example, a better understanding of the functional effects and interactions of the auxiliary microbiome on rumen community assembly and maturation could potentially enable early stage modulation of the adult rumen microbiome.

## Supplementary Material

Early_Microbiome_Supp_Figs_wraf252

S1_table_gtdbtk_representative_bacteria_with_novelty_wraf252

S2_table_gtdbtk_representative_archaea_with_novelty_wraf252

S3_metadata_V2_wraf252

S4_wraf252

S5_Species_Representatives_modules_wraf252

S6_complete_modules_of_novel_prevotella_wraf252

## Data Availability

Out of the 3354 representative metagenome-assembled genomes (MAGs) analyzed in this study, 654 were reconstructed from the current temporal rumen metagenome dataset. These MAGs are publicly available through the GitHub repository Temporal_Genomics_Rumen_MAGs (https://github.com/labmizrahi/Temporal_Genomics_Rumen_MAGs/releases/latest). All other genomes used in this study were obtained from previously published datasets as referenced in the main text.
