# Supplementary material for: Persistent auxiliary microbiome of early novel colonizers in the developing rumen with lasting functional significance
Source: ISME J. 2025 Dec 1;19(1):wraf252. doi: 10.1093/ismejo/wraf252 (PMC12700165; doi:10.1093/ismejo/wraf252)
Supplement: Early_Microbiome_Supp_Figs_wraf252 [file early_microbiome_supp_figs_wraf252.docx]

**Supplementary Figures**


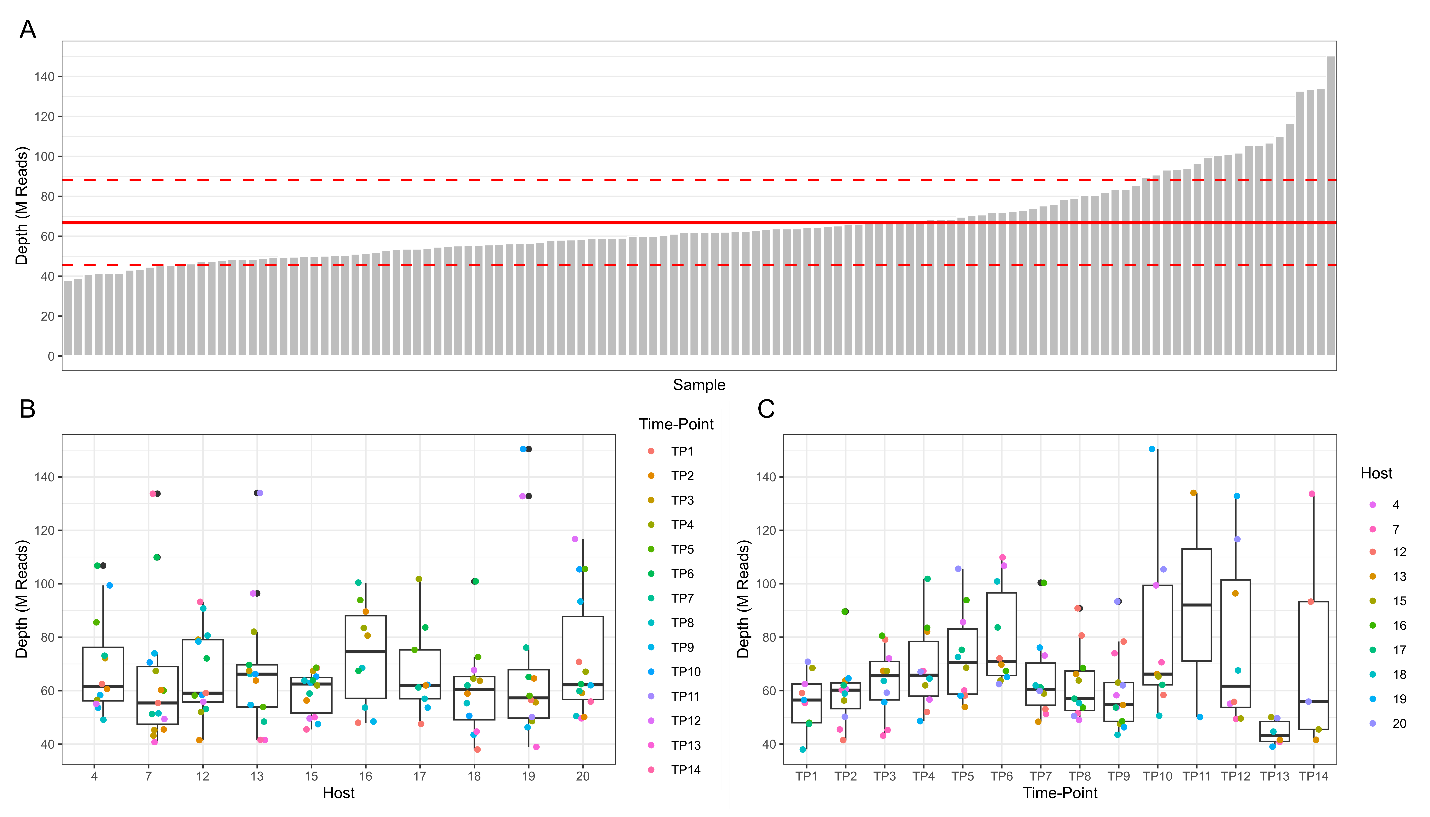


Figure S1. Metagenomic sequencing depth across samples, hosts, and time points.

**A.** Metagenomics sequencing depth (in millions of reads) across all samples, ordered from lowest to highest. Horizontal lines indicate the overall average sequencing depth (solid line) ± standard deviation (dashed lines) across all samples. **B.** Metagenomics sequencing depth (in millions of reads) distribution per host, capturing variability across repeated sequencing of each individual host. C. Metagenomics sequencing depth (in millions of reads) distribution per time-point, summarizing data across all hosts sampled at each respective time point.


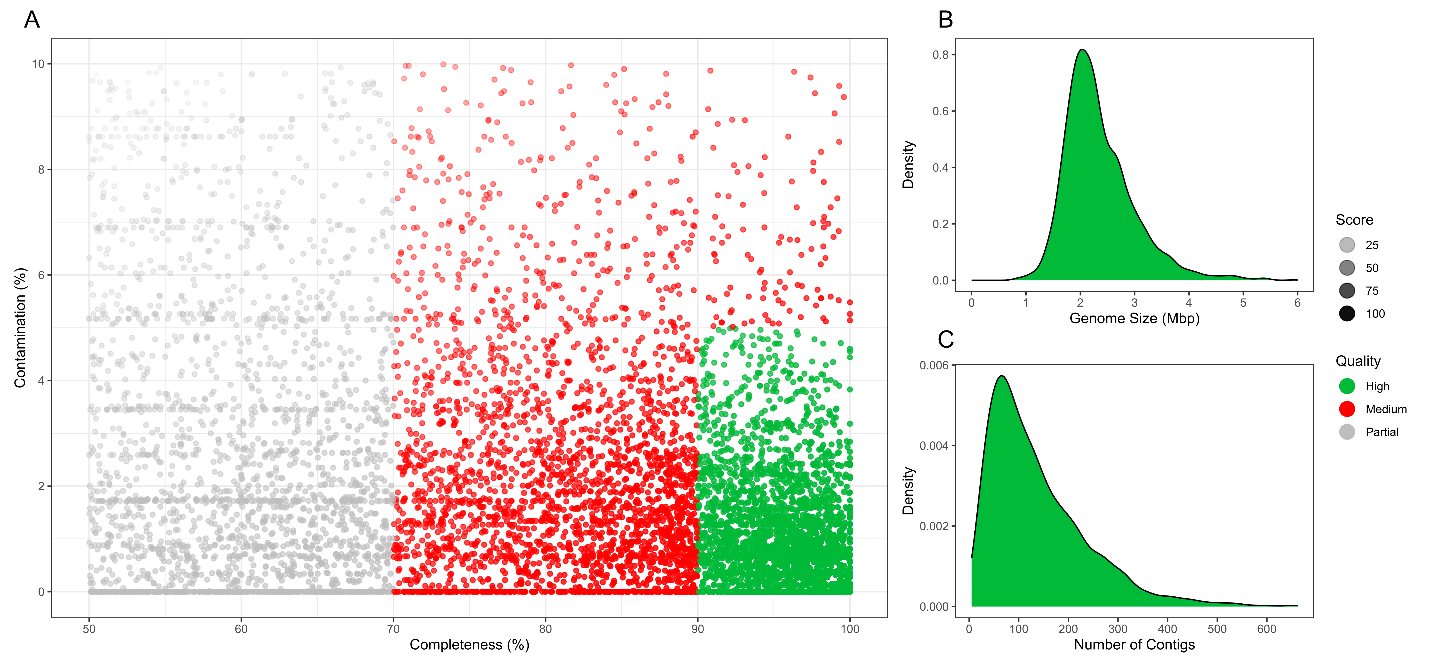


Figure S2. Genome quality metrics and distributions of high-quality metagenome-assembled genomes (MAGs).

A. CheckM completeness and contamination estimates for 7,942 metagenomic-assembled genomes (MAGs) recovered from all samples. The alpha values of the points on the scatter plot correspond to the dRep genome quality score. The reported MAGs meet the following minimum criteria: ≤70% completeness for partial quality genomes, 70% ≤ completeness ≤ 90% or ≥90% completeness and ≥5% contamination for medium quality genomes and ≥90% completeness with ≤5% contamination for high quality genomes. B. the frequency distribution of genome sizes of high quality genomes. C. the frequency distribution of the number of contigs of high quality genomes.


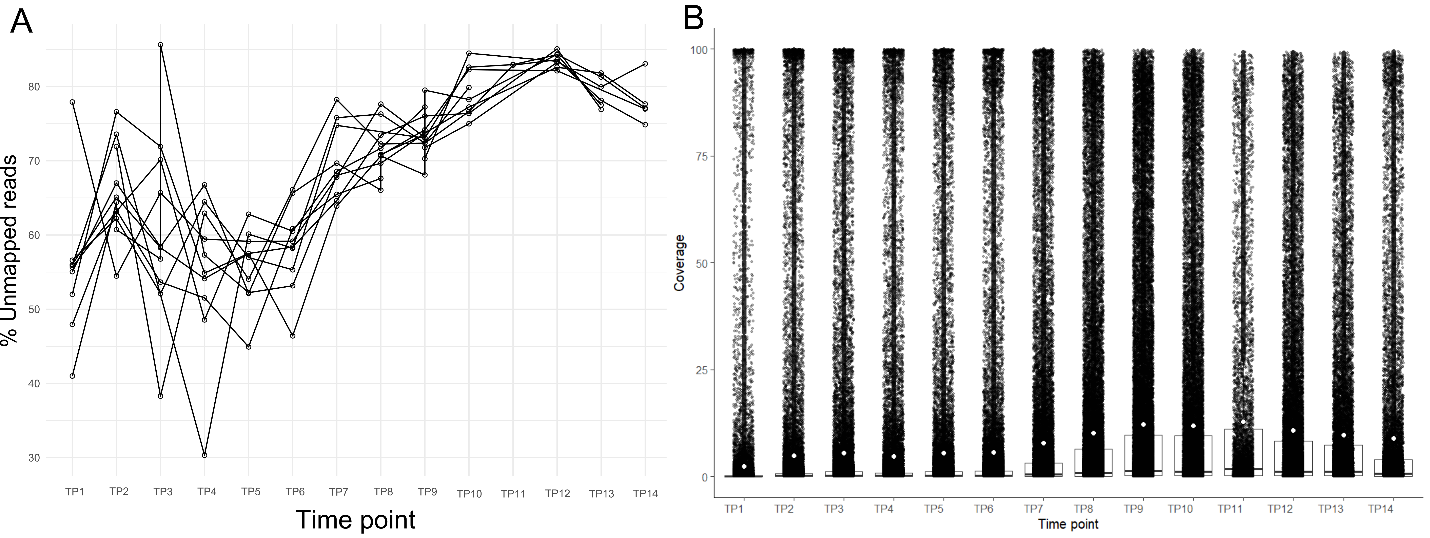


Figure S3. Unmapped read percentages per cow and MAG coverage across time points.

A. Line plots show the percentage of unmapped reads for each cow across the 14 sampled time points (TP1–TP14), with each line representing an individual cow. B. Coverage percentage of 3,354 representative MAGs (y-axis) across time points (x-axis).


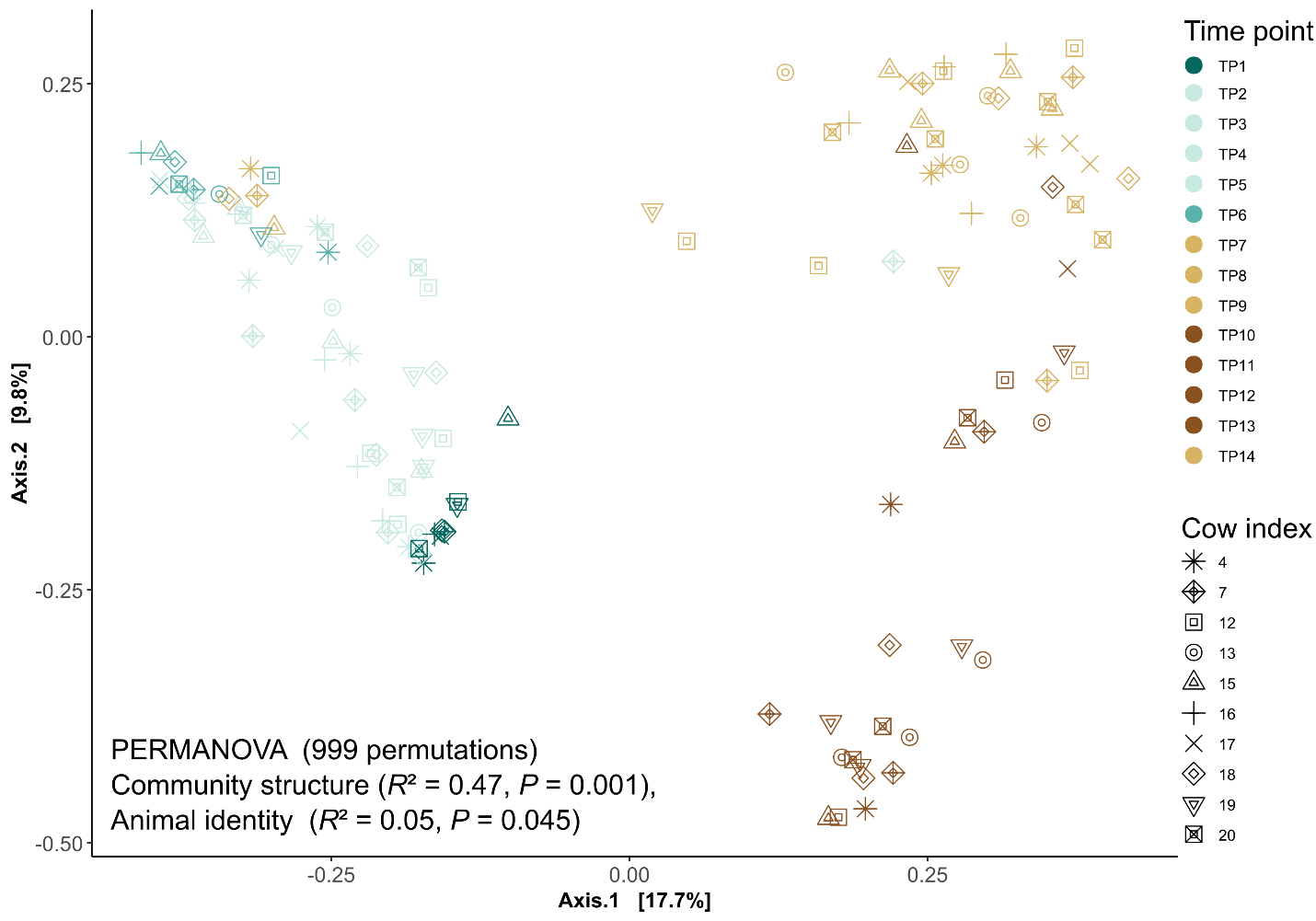


Figure S4. Principal Coordinates Analysis (PCoA) of Bray–Curtis dissimilarities showing clustering of rumen microbial communities by time.

Principal Coordinates Analysis (PCoA) of Bray–Curtis dissimilarities among rumen microbial communities across developmental stages. Community composition was inferred from genome-resolved abundance profiles, calculated by read alignment to MAGs with coverage and normalization procedures as described in the Methods. Each point represents a microbial community sample, with color indicating sampling time point and shape indicating animal ID. Samples cluster strongly by time point, with communities from the same developmental stage converging toward highly similar profiles. In contrast, samples from different time points are clearly separated, showing that temporal progression is the dominant driver of community structure (PERMANOVA, 999 permutations; vegan v2.6-4). Time explained nearly half of the variance (*R*² = 0.47, *P* = 0.001), whereas animal identity accounted for only a small fraction (*R*² = 0.05, *P* = 0.047).


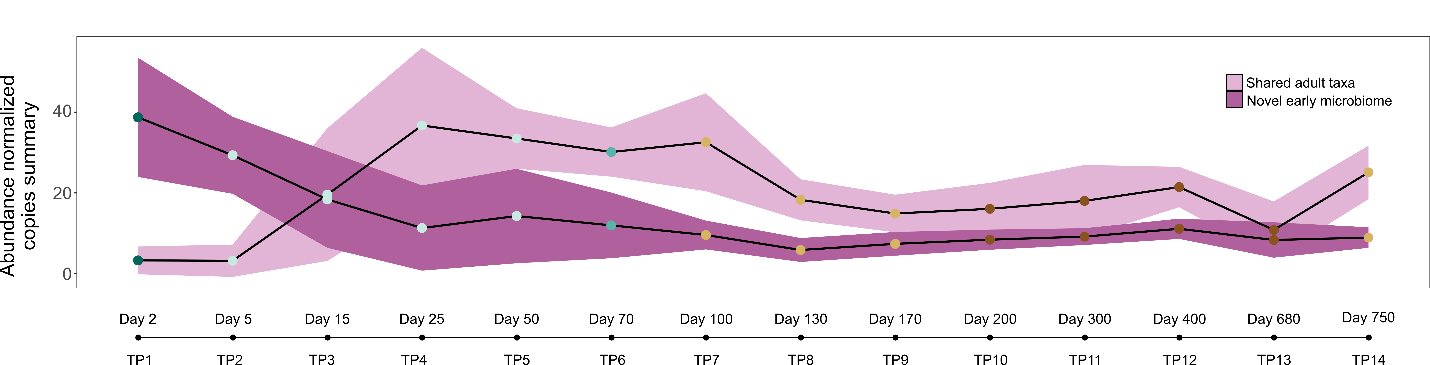


Figure S5. Summary of *ABC transporters* pathway enriched in early auxiliary genomes.

Total abundance-normalized copies (calculated as KO copy number multiplied by genome relative abundance) are presented as mean ± standard deviation for early auxiliary genomes and non auxiliary adult genomes stages. The Y-axis denotes abundance-normalized copy number, and the X-axis represents sampling time points.
